# Monoclonal Antibodies to Intracellular Stages of Cryptosporidium parvum Define Life Cycle Progression *In Vitro*

**DOI:** 10.1128/mSphere.00124-18

**Published:** 2018-05-30

**Authors:** Georgia Wilke, Soumya Ravindran, Lisa Funkhouser-Jones, Jennifer Barks, Qiuling Wang, Kelli L. VanDussen, Thaddeus S. Stappenbeck, Theresa B. Kuhlenschmidt, Mark S. Kuhlenschmidt, L. David Sibley

**Affiliations:** a Department of Molecular Microbiology, Washington University School of Medicine, St. Louis, Missouri, USA; b Department of Pathology and Immunology, Washington University School of Medicine, St. Louis, Missouri, USA; c Department of Pathobiology, University of Illinois College of Veterinary Medicine, Urbana, Illinois, USA; University at Buffalo

**Keywords:** *Cryptosporidium*, cytoskeleton, intracellular parasites, membrane proteins, monoclonal antibodies, sexual development

## Abstract

Among the obstacles hindering *Cryptosporidium* research is the lack of an *in vitro* culture system that supports complete life development and propagation. This major barrier has led to a shortage of widely available anti-*Cryptosporidium* antibodies and a lack of markers for staging developmental progression. Previously developed antibodies against *Cryptosporidium* were raised against extracellular stages or recombinant proteins, leading to antibodies with limited reactivity across the parasite life cycle. Here we sought to create antibodies that recognize novel epitopes that could be used to define intracellular development. We identified a mouse epithelial cell line that supported C. parvum growth, enabling immunization of mice with infected cells to create a bank of monoclonal antibodies (MAbs) against intracellular parasite stages while avoiding the development of host-specific antibodies. From this bank, we identified 12 antibodies with a range of reactivities across the parasite life cycle. Importantly, we identified specific MAbs that can distinguish different life cycle stages, such as trophozoites, merozoites, type I versus II meronts, and macrogamonts. These MAbs provide valuable tools for the *Cryptosporidium* research community and will facilitate future investigation into parasite biology.

**IMPORTANCE** *Cryptosporidium* is a protozoan parasite that causes gastrointestinal disease in humans and animals. Currently, there is a limited array of antibodies available against the parasite, which hinders imaging studies and makes it difficult to visualize the parasite life cycle in different culture systems. In order to alleviate this reagent gap, we created a library of novel antibodies against the intracellular life cycle stages of *Cryptosporidium*. We identified antibodies that recognize specific life cycle stages in distinctive ways, enabling unambiguous description of the parasite life cycle. These MAbs will aid future investigation into *Cryptosporidium* biology and help illuminate growth differences between various culture platforms.

## Abstract (precis)

*Cryptosporidium* is a protozoan parasite that causes gastrointestinal disease in humans and animals. Currently, there is a limited array of antibodies available against the parasite, which hinders imaging studies and makes it difficult to visualize the parasite life cycle in different culture systems. In order to alleviate this reagent gap, we created a library of novel antibodies against the intracellular life cycle stages of *Cryptosporidium*. We identified antibodies that recognize specific life cycle stages in distinctive ways, enabling unambiguous description of the parasite life cycle. These MAbs will aid future investigation into *Cryptosporidium* biology and help illuminate growth differences between various culture platforms.

## INTRODUCTION

*Cryptosporidium* is a genus of protozoan parasites that causes diarrheal disease (cryptosporidiosis) in humans and other animals. Over 20 species are recognized, but the majority of human cases are caused by C. parvum or C. hominis (1). For decades, *Cryptosporidium* was mainly recognized as a cause of chronic diarrhea in patients immunocompromised from HIV/AIDS (2). However, an investigation into the etiologies of diarrheal illnesses in children in Africa and south Asia found that *Cryptosporidium* is second only to rotavirus as a cause of diarrhea in infants in these regions (3). Since diarrheal disease is a significant cause of child mortality (4), this discovery has led to increased interest in cryptosporidiosis and a reexamination of the barriers to studying *Cryptosporidium* (5). The main obstacle hindering research on this parasite is that it cannot be propagated *in vitro* and instead must be passaged through large animals such as calves to generate infectious oocysts. C. parvum can also infect mice, although susceptible mouse models are limited to immunodeficient (6) or neonatal animals (7). Current cell culture models rely on human adenocarcinoma cell lines (e.g., Caco-2 and HCT-8) that do not support complete life cycle development (8). Without the ability to propagate, clonal lines cannot be established and *in vitro* studies are restricted to studying limited rounds of asexual replication and incomplete sexual development. Genetic manipulation is possible if the parasites are passed through immunocompromised mice (9); however, it is not feasible to examine the cellular basis of complex phenotypes without a parallel *in vitro* system for development.

*Cryptosporidium* has a complex life cycle consisting of both an asexual phase (merogony) and a sexual phase (gametogony) that culminates in oocyst formation (10). There are a limited number of antibodies that identify different life cycle stages of *Cryptosporidium*, especially intracellular stages. In part, this limitation is due to the fact that antibodies have typically been generated against extracellular stages such as oocysts (11) and sporozoites (12), and many of these recognize widely conserved epitopes found in intracellular stages (13). For example, it has been reported that epitopes shared on gp60, a glycoprotein involved in parasite adhesion to host cells, and its processed components of gp15 and gp45 are expressed on both sporozoites and merozoites (14–16). Antibodies have also been made against specific proteins (15, 17, 18); however, this method of antibody production does not typically allow for the discovery of novel antigens. Additionally, many antibodies previously raised against *Cryptosporidium* were made in rabbits (15, 18, 19), which is a nonrenewable source of antibody. Collectively, these available reagents do not allow the specific life cycle stages to be clearly delineated with unique markers, confounding attempts to track development during *in vitro* growth.

Our goal was to create a mouse hybridoma bank against *Cryptosporidium* to provide reagents to easily identify life stages, discover new antigens, and provide a renewable reagent source. One barrier to making antibodies against intracellular stages of *Cryptosporidium* is that there is no mouse cell line that supports robust parasite growth (8). Although human adenocarcinoma lines allow for limited intracellular growth, immunization with culture material derived from such heterologous sources would likely generate many host-specific antibodies. Here, we tested primary murine ileal epithelial cells (IECs) and found that C. parvum underwent efficient amplification in these cells. The use of mouse IECs (mIECs) allowed us to immunize mice with infected cell lysates without the risk of generating host-specific antibodies. Using this strategy, we created a hybridoma bank expressing novel monoclonal antibodies (MAbs) against intracellular parasite stages. By examining the timing of expression and patterns of staining, we identified antibodies with distinctive reactivity against specific life cycle stages, such as trophozoites, merozoites, type I and II meronts, and macrogamonts. Collectively, this “antibody toolkit” should lead to a deeper understanding of parasite biology and foster efforts to define conditions for complete development and propagation *in vitro*.

## RESULTS AND DISCUSSION

### Establishing a mouse hybridoma bank against C. parvum.

**FIG 1:**
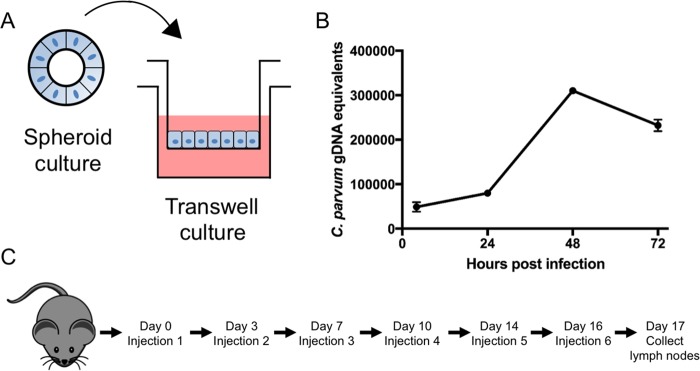
Infecting primary mouse ileal epithelial cells (mIECs) and immunization of mice. (A) Mouse intestinal stem cells were passaged as spheroids. To establish monolayers, spheroids were trypsinized and the cells seeded onto Transwell membranes. (B) Twenty-four hours after plating the monolayer, the cells were infected with oocysts and samples were harvested at intervals to isolate genomic DNA for estimation of growth by qPCR. The data shown are from one experiment but are representative of the growth seen in 3 or more experiments. *n =* 3 Transwells at each time point. Values are means ± standard deviation (SD). (C) Five mice were immunized with infected mIEC lysates, obtained at 48 h postinfection, following a series of 6 footpad injections over 2 weeks. Seventeen days after the initial injection, the mice were sacrificed and the popliteal lymph nodes were isolated.

We initially sought to identify a mouse cell type that would support robust parasite growth *in vitro*. It has been reported that bovine and human isolates of *Cryptosporidium* will grow in mouse L929 fibroblasts (20) and peritoneal macrophages (21), but these cell types are not commonly infected *in vivo*. Instead, *Cryptosporidium* primarily grows in the epithelial cells of the small intestine (22). Recent developments have shown that mouse intestinal stem cells can be maintained in culture as spheroids using media containing a cocktail of specific growth factors (23). When these stem cells are grown as monolayers on Transwells, they develop into polarized epithelial monolayers that include enteroendocrine cells, Paneth cells, mucin-producing goblet cells, and enterocytes (24). As shown previously, these different lineages are present in the monolayers at rates similar to what is found in the mouse intestine (24). Such monolayers could provide a much more natural niche for growth of C. parvum than typical transformed cell lines. Consequently, we tested primary mouse ileal epithelial cells (mIECs) that were differentiated from intestinal stem cells for their ability to support C. parvum growth *in vitro*. C. parvum was able to achieve high levels of amplification over a 3-day period in mIECs grown in Transwells as shown by quantitative PCR (qPCR) (Fig. 1).

**FIG 2:**
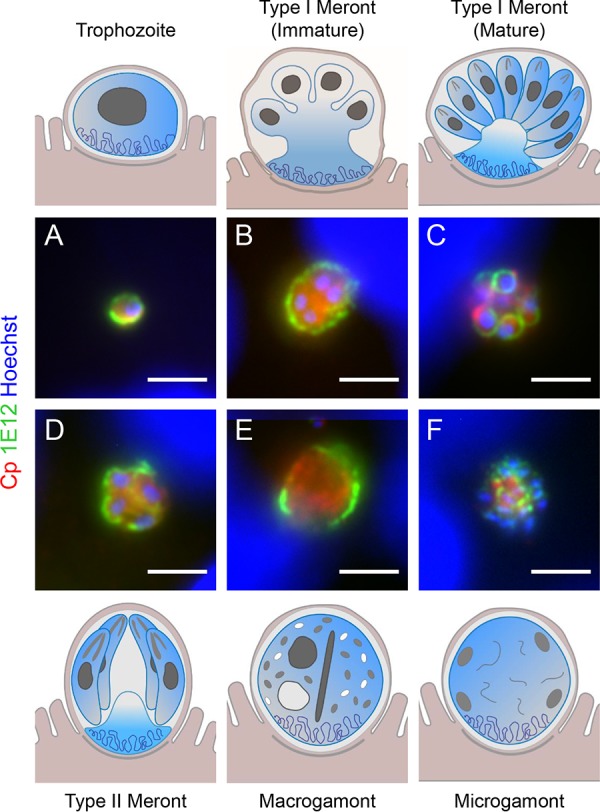
MAb 1E12 recognizes the parasite membrane of all life cycle stages, as shown by the cartoons at the top and bottom (courtesy of Laura Kyro, reproduced with permission). Infected HCT-8 cells were fixed and stained with MAb 1E12 (green), rabbit anti-RH (Cp [red]) to detect C. parvum, and Hoechst to detect DNA. Samples from 4 h postinfection were used to image trophozoites (A) and immature (B) and mature (C) type I meronts, 32 h postinfection for type II meronts (D), or 48 h postinfection for macrogamonts (E) and microgamonts (F). Scale bars = 3 µm.

Having successfully identified a mouse cell line that supported robust development of C. parvum, we immunized mice with infected mIEC lysates, performed a fusion, and screened the resulting hybridomas by microscopy to identify novel antibodies to C. parvum. Following sequential subcloning, individual monoclonal antibodies (MAbs) were screened on infected mIECs and HCT-8 cells to investigate their reactivity against different life cycle stages of C. parvum. To monitor C. parvum infection, we stained the monolayers with a rabbit polyclonal antibody raised against the Toxoplasma gondii strain RH that was broadly cross-reactive to C. parvum antigens based on Western blotting (see Fig. S1 in the supplemental material). As shown in Fig. 2, this anti-RH antibody (labeled “Cp” in figures) recognized both asexual and sexual stages of C. parvum by immunofluorescence (immunofluorescent antibody assay [IFA]).

10.1128/mSphere.00124-18.1 FIG S1  Antibody reactivity by Western blotting. (A) Western blot analysis of samples separated by 10% SDS-PAGE. Lanes show loading of samples as follows: oocysts at 1 × 10^4^ (lane 2) and 2 × 10^4^ (lane 3) and sporozoites at 1 × 10^6^ (lane 4) and 2 × 10^6^ (lane 5). The blot was probed with anti-RH antibody at 1:500, and goat anti-rabbit IR dye 800CW was used at a 1:10,000 dilution. (B) Western blot analysis of samples separated by 10% SDS-PAGE. Each lane was loaded with 4 × 10^6^ sporozoites. The blot was probed with the respective monoclonal antibodies at 1:250 each and a secondary goat anti-mouse antibody conjugated with HRP at 1:10,000. Detection was done with an ECL Prime Western kit at an exposure time of 2 min. Download FIG S1, TIF file, 1.3 MB. Copyright © 2018 Wilke et al. 2018 Wilke et al. This content is distributed under the terms of the Creative Commons Attribution 4.0 International license.

Starting from a total of 31 MAbs, we identified 12 antibodies with distinctive staining patterns (Table 1). Other MAbs with positive reactivity showed patterns similar to those described here and therefore they were not pursued further. Overall, most MAbs reacted to multiple stages (Table 1; see Fig. S2 and S3 in the supplemental material), suggesting that they recognize epitopes that are expressed broadly across the intracellular life cycle. We identified one antibody, 1E12, that recognized every life stage, excluding oocysts (Table 1 and Fig. S2 and S3). Based on its reactivity pattern, 1E12 recognizes an epitope associated with the membrane of all life cycle stages (Fig. 2). Due to its broad spectrum and membrane pattern of reactivity, 1E12 can be used to identify specific stages based on appearance, size, and number of nuclei. For example, 1E12 can be used to distinguished type I meronts, which have 4 nuclei but a single cytoplasmic mass (Fig. 2B), from type II meronts, which have four nuclei but have separated into 4 distinct merozoites (Fig. 2D). MAb 1E12 also stains the surface of type I merozoites (Fig. 2C), allowing mature type I meronts to be clearly distinguished from immature type I meronts. MAb 1E12 also recognized the membrane of macrogamonts (Fig. 2E) and one end of the small microgametocytes within the microgamont (Fig. 2F).

**TABLE 1:**
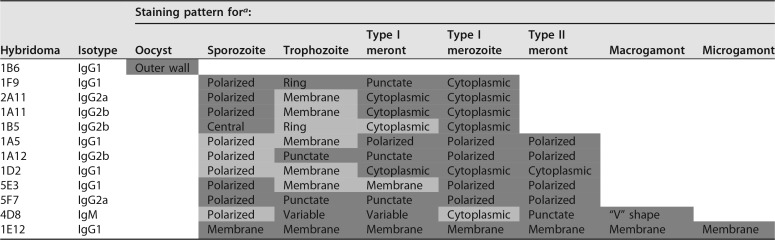
Staining patterns of mouse monoclonal antibodies against C. parvum

a Staining patterns are based on reactivity to infected mIECs (Fig. S2 and 3) and infected HCT-8 cells (images in the main figures) and reactivity on sporozoites (Fig. S4). The different levels of shading denote strength of reactivity, with dark gray corresponding to strong staining and light gray reflecting weaker, positive staining. Blank table cells indicate no reactivity.

10.1128/mSphere.00124-18.2 FIG S2  Staining patterns of monoclonal antibodies in infected mIECs. mIECs were infected with C. parvum oocysts and fixed 24 h after infection. Cells were stained with the specified mouse MAbs and rabbit anti-RH to detect C. parvum (Cp). The secondary antibodies used were goat anti-mouse conjugated with Alexa Fluor 488 and goat anti-rabbit conjugated with Alexa Fluor 594. The life cycle stage cartoons at the top were created by Laura Kyro and are reproduced with permission. Scale bars = 5 µm. Download FIG S2, TIF file, 2.5 MB. Copyright © 2018 Wilke et al. 2018 Wilke et al. This content is distributed under the terms of the Creative Commons Attribution 4.0 International license.

10.1128/mSphere.00124-18.3 FIG S3  Staining patterns of monoclonal antibodies in infected mIECs. mIECs were infected with C. parvum oocysts and fixed 24 h after infection. Cells were stained with the specified mouse MAbs and rabbit anti-RH to detect C. parvum (Cp). The secondary antibodies used were goat anti-mouse conjugated with Alexa Fluor 488 and goat anti-rabbit conjugated with Alexa Fluor 594. The life cycle stage cartoons at the top were created by Laura Kyro and are reproduced with permission. Scale bars = 5 µm. Download FIG S3, TIF file, 2.2 MB. Copyright © 2018 Wilke et al. 2018 Wilke et al. This content is distributed under the terms of the Creative Commons Attribution 4.0 International license.

MAb 1E12 has great utility as a “pan-crypto” antibody; it can be used to easily assess the ability of different cell culture platforms to support *Cryptosporidium* growth as it recognized all the major stages, which are distinguishable by their surface morphology. Other antibodies identified through the screen did not have this broad reactivity but rather identified specific life cycle stages in distinguishable patterns, making them useful for understanding life cycle progression in different culture systems. These antibodies are described in detail below.

### MAb 1B6 recognizes the oocyst outer wall.

**FIG 3:**
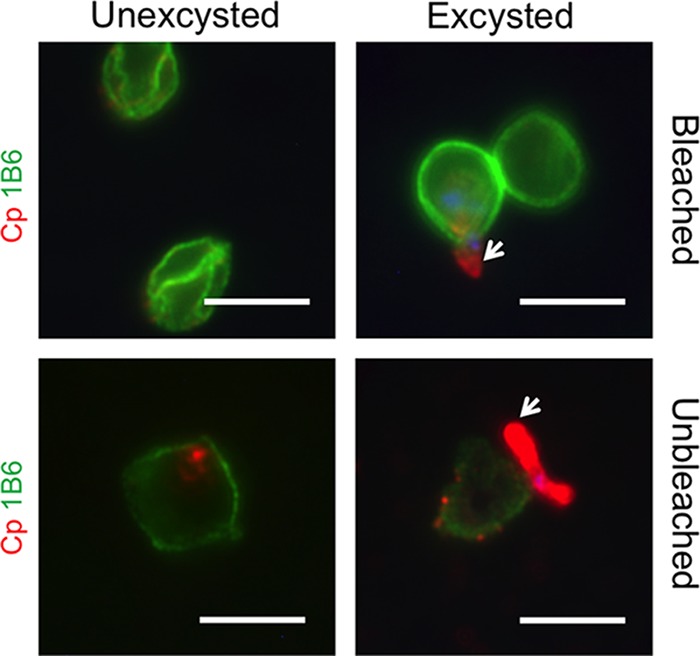
MAb 1B6 antibody stains the outer oocyst wall. Oocysts were either left unbleached or were bleached for 10 min in a 40% bleach solution and then were either left unexcysted or were excysted in a 0.75% sodium taurocholate solution for 60 min at 37°C. Oocysts were then plated on coverslips coated with poly-l-lysine. Samples were fixed and stained with 1B6 (green) and rabbit anti-RH (Cp [red]) to detect C. parvum. Arrows indicate sporozoites. Scale bars = 5 µm.

Only one antibody from the hybridoma library was specific for a single stage; MAb 1B6 only recognized oocysts and did not show any reactivity toward asexual (Fig. S2 and S3) or sexual (Table 1) stages. MAb 1B6 detected residual oocysts from the inoculum that were present in mIECs (Fig. S1). MAb 1B6 stained the oocyst wall in a continuous pattern (Fig. 3), similar to Crypt-a-glo, a commercial reagent that is an oocyst-specific monoclonal antibody (Waterborne, Inc.) (25). MAb 1B6 equally recognized oocysts that were either left unstimulated or stimulated to excyst (Fig. 3), suggesting it recognizes an epitope on the outer wall of the oocyst. MAb 1B6 preferentially stained bleached oocysts (Fig. 3), a process that removes the outer veil (26). Based on this result, it is likely that bleach treatment removes the veil and exposes the 1B6 epitope on the outer wall. Interestingly, 1B6 did not stain macrogamonts (Table 1), unlike the previously described MAb, OW50, which also reacts to an outer oocyst wall protein and stains wall-forming bodies in mature macrogamonts (27).

### MAbs 1B5 and 1F9 have distinctive trophozoite-specific patterns.

**FIG 4:**
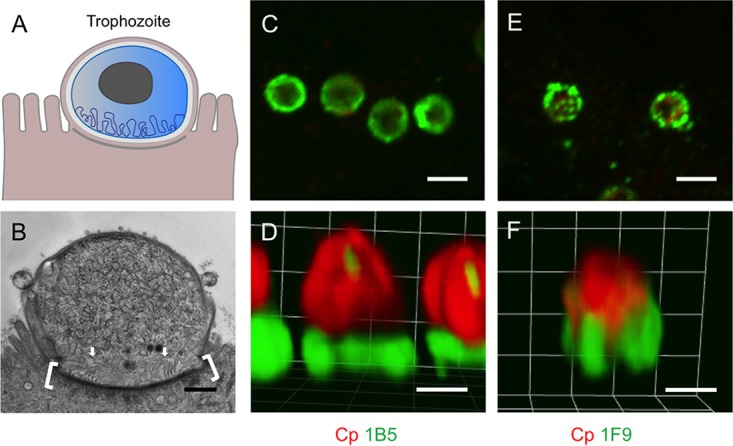
Antibodies that recognize unique patterns in trophozoites. (A) Cartoon representing the trophozoite stage (courtesy of Laura Kyro, reproduced with permission). (B) Transmission electron micrograph of trophozoite growing in mIEC 24 h postinfection. The actin-rich host cell cytoskeleton is denoted by brackets. The membrane-rich feeder organelle is indicated by arrows. Scale bar = 500 nm. (C and D) Pattern of staining with MAb 1B5 (green) and rabbit anti-RH (Cp [red]) to detect C. parvum. (E and F) Pattern of staining for MAb 1F9 and rabbit-anti-RH (Cp [red]) to detect C. parvum. For panels C to F, HCT-8 cells were infected with oocysts and fixed and stained 24 h postinfection. Panels C and E show single z-slices acquired by laser scanning confocal microscopy, Panels D and F show the 3D rendering of a full z-stack. Scale bars in panels C and E = 2 µm; scale bars in panels D and F = 1 µm.

Although many of the antibodies generated here react to multiple life cycle stages, they still provide markers for defining development due to the unique patterns of staining they detect in specific stages. For example, MAbs 1B5 and 1F9 detect a distinctive “donut-shaped” pattern in trophozoites during the initial stages of asexual development (Fig. 4C and E). Although both MAbs have a circular staining pattern on trophozoites, 1B5 staining has a smooth, continuous appearance, while 1F9 is punctate, implying the two antibodies recognize different epitopes. While 1F9 did not show reactivity by Western blotting, 1B5 recognized a 230-kDa product in sporozoites (Fig. S1).

When images of trophozoites were acquired by laser scanning confocal microscopy and the z-stacks were rendered in three dimensions (3D), the resulting images revealed that the epitope recognized by 1B5 is mostly confined to the base of the parasite, where it contacts the host cell, while 1F9 staining begins at the base and extends up the side of the vacuole or parasite membrane (Fig. 4D and F). Because these antibodies have a polarized recognition pattern in sporozoites (see Fig. S5 in the supplemental material), their targets likely relocalize following invasion to this interface during initial trophozoite growth.

The host-parasite interface is where the host cytoskeleton is reorganized into an actin-rich pedestal during intracellular development (28–30). When infected HCT-8 cells were stained with fluorescently labeled phalloidin to visualize this actin pedestal, labeling with 1B5 and 1F9 revealed that both MAbs formed a ring around the nascent pedestal in trophozoites (Fig. S4A). Examination of z-slices generated by laser scanning confocal microscopy revealed that the ring formed by 1B5 is within the same plane as the pedestal, whereas the 1F9-labeled ring is positioned above the pedestal (Fig. S4A). As trophozoites develop into meronts, the epitopes recognized by 1B5 and 1F9 were redistributed, leading to more diffuse labeling that was no longer confined to the base of the parasite (Table 1, Fig. S2, and Fig. S4B).

10.1128/mSphere.00124-18.4 FIG S4  Distribution of 1B5 and 1F9 staining in trophozoites and meronts relative to the actin pedestal. (A) HCT-8 cells were infected with oocysts and fixed 2 h postinfection to detect trophozoites. Cells were stained with phalloidin, MAb 1F9 or 1B5, and rabbit anti-RH to detect C. parvum (Cp). The secondary antibodies used were goat anti-mouse conjugated with Alexa Fluor 555 and goat anti-rabbit conjugated with Alexa Fluor 647. In each row, the panels to the left are the 3D-rendered images from a z-stack acquired by laser scanning confocal microscopy and the additional image to the right is a single z-slice from the z-stack. Scale bars = 3 µm. (B) HCT-8 cells were infected with oocysts and fixed 24 h postinfection to detect meronts. Cells were stained with phalloidin, 1F9 or 1B5, and rabbit anti-RH to detect C. parvum (Cp). The secondary antibodies used were the same as for panel A. In each row, the three panels to the left are the 3D-rendered images from a z-stack acquired by laser scanning confocal microscopy and the additional image to the right is a single z-slice from the z-stack. Scale bars = 3 µm. Download FIG S4, TIF file, 1.7 MB. Copyright © 2018 Wilke et al. 2018 Wilke et al. This content is distributed under the terms of the Creative Commons Attribution 4.0 International license.

10.1128/mSphere.00124-18.5 FIG S5  Monoclonal antibodies that recognize sporozoites. Oocysts were excysted and plated onto PLL-coated coverslips. Sporozoites were fixed and stained with the specified mouse MAbs and rabbit anti-RH to detect C. parvum (Cp). For 1B5 staining, samples were permeabilized with 0.1% Triton X-100. The secondary antibodies used were goat anti-mouse conjugated with Alexa Fluor 488 and goat anti-rabbit conjugated with Alexa Fluor 594. Scale bars = 5 µm. Download FIG S5, TIF file, 1.5 MB. Copyright © 2018 Wilke et al. 2018 Wilke et al. This content is distributed under the terms of the Creative Commons Attribution 4.0 International license.

The host-parasite interface is also the site where the parasite elaborates a membrane-rich feeder organelle (31) (Fig. 4A). The nature of this feeder organelle is uncertain, although the genomes of C. parvum (32) and C. hominis (33) contain a large number of transporters, and it has been speculated that this interface is responsible for transport of nutrients from the host (34). Consistent with this, a C. parvum ATP-binding cassette protein that shares features with transporters has previously been localized to this interface (18). Attempts to localize the epitopes recognized by 1B5 or 1F9 using immunoelectron microscopy were not successful; however, based on their localization pattern in trophozoites by fluorescence microscopy, it is possible that the targets recognized by either MAb are involved in the reorganization of the host cytoskeleton and the generation of the feeder organelle. Hence, these MAbs may facilitate future investigation into these two developmental processes.

### MAb 5E3 recognizes mature type I merozoites in a polarized manner.

**FIG 5:**
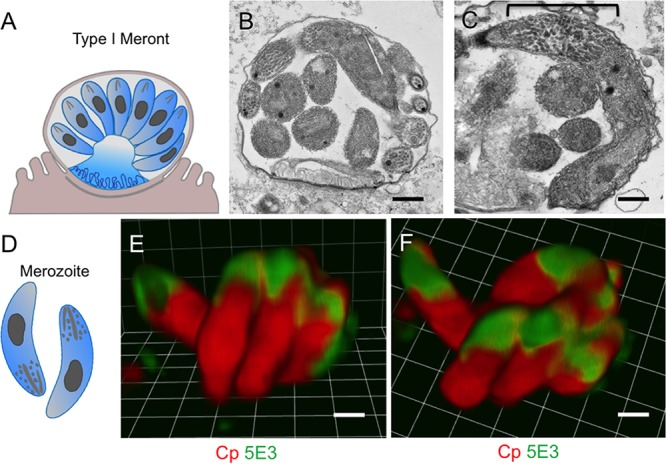
MAb 5E3 recognizes the apical end of merozoites. (A and D) Cartoons representing the type I meront and merozoite, respectively (courtesy of Laura Kyro, reproduced with permission). (B and C) Transmission electron micrograph of a mature meront in HCT-8 cells 24 h postinfection. Brackets denote micronemes at the apical end of the merozoite. Scale bars = 500 nm. (E and F) HCT-8 cells were infected with oocysts, fixed at 24 h postinfection, and stained with MAb 5E3 (green) and rabbit-anti-RH (Cp [red]) to detect C. parvum. (E and F) Side (E) and top (F) view images of 3D-rendered images from a z-stack acquired by laser scanning confocal microscopy. Scale bars = 1 µm.

Moving forward in the parasite life cycle, the parasite progresses through asexual development to replicate mitotically and generate 8 mature merozoites within type I meronts (Fig. 5A). Transmission electron microscopy reveals that the apical end of merozoites in mature type I meronts is characterized by many small secretory vesicles corresponding to micronemes and cross sections of rhoptries (Fig. 5B and C), similar to what is seen in sporozoites (35). Four MAbs (1A5, 1A12, 5E3, and 5F7) recognized both type I and type II merozoites in a distinctive polarized pattern reminiscent of this apical specialization (Table 1), and these MAbs, along with others, also stain the apical end of sporozoites (Fig. S5). An example of this polarized staining pattern is shown for 5E3 staining of fully developed merozoites in mature type I meronts (Fig. 5E and F and Fig. S3). MAb 5E3 was also able to recognize immature type I meronts, where the epitope is fainter and distributed along the membrane (Table 1) from mature type I meronts that contain 8 merozoites, which demonstrate strong polarized staining (Fig. S3). Three-dimensional rendering of z-stacks acquired by laser scanning confocal microscopy revealed that the epitope recognized by 5E3 is concentrated at one end of the parasite (Fig. 5E and F). This polarized pattern is similar to that of a previously published mouse monoclonal antibody, 4E9, which stains the apical end of sporozoites and also recognizes trails of protein shed by the sporozoite during gliding motility (12). Attempts to label the apical end of sporozoites directly with 5E3 by immunoelectron microscopy were unsuccessful, but based on its recognition pattern by fluorescence microscopy, it is likely that MAb 5E3 and other antibodies that stain in a highly polarized pattern (Table 1) recognize components of apical secretory organelles, which are implicated in host cell attachment and invasion (36, 37).

### Antibodies that distinguish type I from type II meronts.

**FIG 6:**
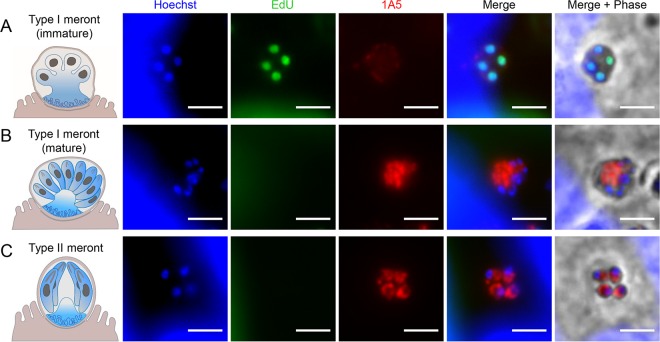
MAb 1A5 recognizes mature type I and type II meronts but not immature type I meronts. Cartoons representing the type I and II meronts were created by Laura Kyro and are reproduced with permission. (A) Staining of immature type I meronts defined by four EdU-positive nuclei and lack of defined merozoites by phase-contrast (Merge + Phase). (B) Staining of mature type I meronts defined by a lack of EdU staining and the presence of defined merozoites by phase-contrast microscopy (Merge + Phase). (C) Staining of type II meronts as defined by four nuclei that lack EdU staining as well as the presence of 4 individual merozoites by phase-contrast microscopy (Merge + Phase). Infected HCT-8 cells were incubated with the thymidine analog EdU for 2 h starting 30 h postinfection and then were fixed and treated with an EdU Click-It 488 labeling kit (green) and stained with MAb 1A5 (red) and Hoechst DNA stain (blue). Scale bars = 3 µm.

The life cycle of *Cryptosporidium* proceeds through two rounds of merogony, the first of which culminates in eight merozoites (type I), while the second terminates with four merozoites (type II) (31). Because the type I merogony cycle also proceeds through a 4-nucleus stage, it can be difficult to distinguish these immature type I stages from mature type II stages based on the number of nuclei alone. To identify antibodies that recognize type II meronts, we established a method to reliably distinguish type II meronts, which contain 4 nuclei within mature merozoites, from “early stage” or immature type I meronts, which can also contain 4 nuclei. To distinguish between these stages, we pulse-labeled parasites growing in HCT-8 cells using 5-ethynyl-2′-deoxyuridine (EdU), which labels replicating DNA (9), and detected incorporation fluorescently using click labeling (see Materials and Methods). By adding EdU in specific intervals after infection, we identified actively replicating stages that were further defined by nuclear morphology and number. Type II meronts were most commonly detected from 30 to 32 h postinfection. When EdU was added during this 2-h interval, type II meronts were identified by the presence of 4 nuclei that lacked EdU positivity, as they were mature and no longer replicating, whereas 4-nucleus type I meronts were EdU positive because they were still actively replicating (Fig. 6).

Based on differential EdU labeling, we identified several antibodies that recognized type II meronts with different staining patterns (Table 1). For example, MAb 1A5 did not stain immature type 1 meronts (Fig. 6A) but did stain mature type I (Fig. 6B) and type II (Fig. 6C) meronts. Because of this pattern of reactivity, MAb 1A5 can be used to positively identify type II meronts from immature type I meronts, which also contain 4 nuclei. By Western blotting, 1A5 recognized a 125-kDa band (Fig. S1), and this protein serves as a marker of mature merozoites, whether they are type I or II.

Despite their superficial similarity, merozoites produced by these two rounds of merogony have different fates. Type I merozoites are thought to reinitiate multiple rounds of asexual replication, while type II merozoites are thought to give rise to gamonts (10). Unfortunately, we did not identify any MAbs that only stain type II meronts, nor have such reagents been described previously, although differences have been detected by ultrastructure (31). Consequently, the ability to track type II meronts using MAbs like 1A5 combined with EdU staining provides a convenient means of monitoring development of stages that are committed to undergo sexual development.

### Antibodies that distinguish sexual stage development.

**FIG 7:**
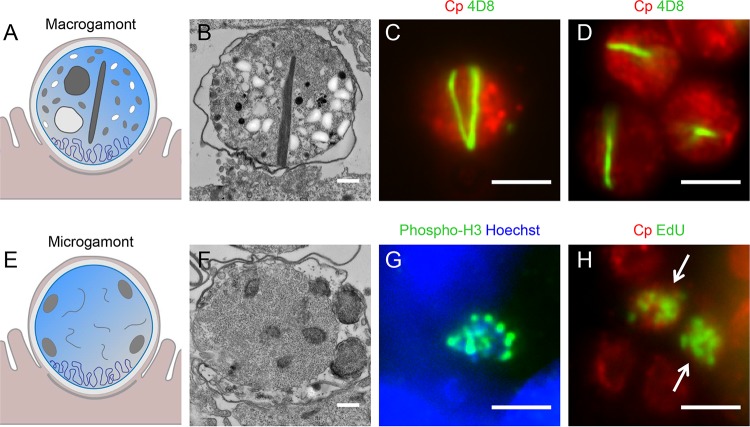
Antibodies with reactivity to C. parvum sexual stages. (A and E) Cartoons representing the macrogamont and microgamont, respectively (courtesy of Laura Kyro, reproduced with permission). (B) Transmission electron micrograph of the macrogamont within an HCT-8 cell 48 h postinfection. Scale bar = 500 nm. (C and D) Infected HCT-8 cells were fixed and stained 72 h postinfection with rabbit-anti-RH (Cp [red]) to detect C. parvum and MAb 4D8 (green). Scale bars = 3 µm. (F) Transmission electron micrograph of the microgamont within an HCT-8 cell 48 h postinfection. Scale bar = 500 nm. (G) Infected HCT-8 cells were fixed and stained 72 h postinfection with an anti-phosphohistone H3 (Ser10) antibody (green). Scale bar = 3 µm. (H) Infected HCT-8 cells were incubated with the thymidine analog EdU for 2 h starting 46 h postinfection before fixation. Cells were treated with an EdU Click-It 488 labeling kit (green) to detect microgametocyte nuclei undergoing replication (arrows) and stained with rabbit-anti-RH (Cp [red]) to detect C. parvum. Scale bar = 3 µm.

Merozoites released by type II meronts are thought to give rise to micro- and macrogamonts, which eventually undergo fertilization to form oocysts, although this last step does not occur efficiently *in vitro*. The majority of MAbs studied here did not recognize sexual stages, with two exceptions (Table 1). For example, MAbs 1A5 and 1B5 broadly stain asexual stages but fail to stain either micro- or macrogamonts (Table 1). The membrane-reactive MAb 1E12 did stain the membranes of both microgamonts and macrogamonts (Fig. 2 and Table 1), although the staining of macrogamonts did not provide a unique recognition pattern that differentiated it from asexual stages such as meronts. In contrast, MAb 4D8 showed a characteristic “V” (Fig. 7C) or line pattern (Fig. 7D) in macrogamonts (Table 1), a reactivity pattern not reported in any published studies with other *Cryptosporidium* antibodies. When examined by transmission electron microscopy, macrogamonts were found to contain a prominent striated fiber running through the center of the cytoplasm (Fig. 7B). This striated fiber is not seen in all thin sections, possibly explaining why some electron microscopy studies of *in vivo* infection do not mention the fiber at all (31), and only one published study contains an image of a macrogamont with a fiber, although it is not described in detail (38). Attempts to label the fiber directly with 4D8 by immunoelectron microscopy were not successful, so while we cannot definitively say 4D8 recognizes this structure, it seems highly likely based on its appearance. The molecular nature of this striated fiber is also uncertain, as the streamlined genome of C. parvum contains homologs for tubulin, actin, and several actin-related proteins (http://cryptoDB.org). The genome also contains orthologues of articulins, which are known in other apicomplexans as inner membrane complex proteins (IMCs) (39), although they are not annotated as such in C. parvum (http://cryptoDB.org). The striated fiber observed in macrogamonts does not resemble microtubules that are typically found apically or the meshwork of IMC proteins that are localized under the membrane of motile stages (39). Hence, this striated fiber may represent a novel assemblage of actin filaments, or a novel cytoskeletal element that imparts some form of structural integrity within macrogamonts. Currently, macrogamonts are recognized by their size and diffuse nucleus. In contrast, the staining pattern of 4D8 provides a definitive marker for this stage based on the strongly striated pattern, while it stains other stages much more diffusely (Fig. S3).

We did not identify any antibodies that specifically stain microgamonts, although they are recognizable by their many small nuclei, which number 16 in mature microgamonts (Fig. 7). These small, replicating nuclei were easily visualized using a commercially available anti-phosphohistone H3 antibody (Fig. 7G), which stains DNA during mitosis (40), or through the incorporation of EdU during DNA replication (Fig. 7H).

### Conclusions.

Previous studies on the development of C. parvum* in vitro* have been hampered both by the lack of an efficient *in vitro* system for propagation and by the lack of specific reagents to stage development. We have taken advantage of developments in stem cell biology to propagate C. parvum in mouse IECs, which more closely resemble the intestinal cells that support growth *in vivo* compared to adenocarcinoma cell lines. Although attempts to achieve complete development of C. parvum in mIECs are ongoing, here we have used this system to generate antigens for production of novel MAbs to intracellular stages. This approach has been effective at generating reagents that define stage-specific patterns of expression, which greatly enhances our ability to define specific stages during *in vitro* growth. Collectively these reagents should be useful for future studies to (i) define developmental progression during *in vitro* culture, (ii) identify conditions necessary to support complete development *in vitro*, and (iii) pinpoint the stages that are susceptible to chemotherapy, thereby supporting efforts at target identification.

## MATERIALS AND METHODS

### Ethics statement.

Animal studies were conducted according to the *Public Health Service Policy on Humane Care and Use of Laboratory Animals* (Office of Laboratory Animal Welfare, National Institutes of Health, Bethesda, MD). Animals were maintained in Association for Assessment and Accreditation of Laboratory Animal Care-approved facilities. Animal studies were approved by the Institutional Animal Studies Committee at the School of Medicine, Washington University in St. Louis.

### Adenocarcinoma cell culture.

Human ileocecal adenocarcinoma cells (HCT-8; ATCC CCL-244) were maintained in RPMI 1640 medium (Gibco, ATCC modification) supplemented with 10% fetal bovine serum (FBS). Human colorectal adenocarcinoma cells (Caco-2; ATCC HTB-37) were maintained in minimum essential medium (Corning CellGro) supplemented with 20% FBS. Cell lines were tested for the presence of mycoplasma and confirmed negative with the e-Myco plus Mycoplasma PCR detection kit (Boca Scientific).

### 3D spheroid cell culture.

Primary ileal epithelial stem cells isolated from 8- to 10-week-old C57BL/6 mice were obtained from the laboratory of Thad Stappenbeck, Washington University in St. Louis. Ileal stem cells were expanded and maintained as 3D spheroid cultures in Matrigel (BD Biosciences), as described previously (41). Spheroid cultures were grown in 50% L-WRN cell-derived conditioned medium (CM) containing 10 µM Y-27632 (ROCK inhibitor; Tocris Bioscience). The medium was changed every 2 days, and the cells were passaged every 3 days in a 1:6 split.

### Formation of Transwell monolayers.

To form monolayers, spheroids from 3-day-old stem cell cultures were recovered from Matrigel and dissociated with trypsin as described previously (24). Transwells (polyester membrane, 0.4-µm pore; Corning Costar) were prepared for cell seeding by coating the upper compartment with 100 µl of a 1:40 dilution of Matrigel for 20 min at 37°C. Excess Matrigel was aspirated off the membrane, and approximately 2 × 10^5^ cells, diluted in 100 µl CM with 10 µM Y-27632, were seeded onto the coated membrane. Medium (700 µl of CM with Y-27632) was added to the bottom compartment of the Transwell. About 24 h after seeding, the medium in the top and bottom compartments of the Transwell was changed to 0% CM, referred to as “primary medium” (consisting of advanced Dulbecco’s modified Eagle’s medium [DMEM]–Ham’s F-12 containing 20% fetal bovine serum, 100 U of penicillin, 0.1 mg/ml streptomycin, and 2 mM l-glutamine [Sigma]). Monolayers were infected with oocysts 24 h after seeding.

### Oocyst preparation and excystation.

Oocysts were provided by the Kuhlenschmidt lab (University of Illinois at Urbana Champaign). The AUCP-1 isolate of C. parvum was maintained in male Holstein calves, and oocysts were purified as described previously (42). Oocysts were stored at 4°C in 50 mM Tris–10 mM EDTA (pH 7.2). Before infection, 1 × 10^8^ purified oocysts were diluted into 1 ml of Dulbecco’s phosphate-buffered saline (DPBS; Corning Cellgro) and treated with 1 ml of 40% bleach (commercial laundry bleach containing 8.25% sodium hypochlorite) for 10 min on ice. Oocysts were then washed 4 times in DPBS containing 1% (wt/vol) bovine serum albumin (BSA; Sigma) and resuspended in 1 ml DPBS with 1% BSA. For some experiments, oocysts were excysted prior to infection by incubating the oocysts with 0.75% (wt/vol) sodium taurocholate (Sigma) at 37°C for 60 min. Excysted oocysts were washed once with cell medium prior to being added to cells.

For sporozoite and oocyst labeling experiments, coverslips were coated with poly-l-lysine (PLL; Sigma) overnight at room temperature. After aspirating the PLL and allowing the coverslips to dry, unexcysted oocysts or a mixture of excysted oocysts and sporozoites were added to the coverslips and allowed to settle for 20 min. Unbleached oocysts were washed three times with sterile DPBS before being plated onto coverslips. The oocysts or sporozoites were fixed in 4% formaldehyde, permeabilized with 0.05% saponin (except where stated), and stained.

### Growth of C. parvum in Transwell mIEC monolayers.

At 24 h after plating, mIECs were infected with 2 × 10^6^ oocysts diluted in primary medium added to the top compartment of the Transwell. The medium was changed in the top and bottom compartments of the Transwell daily during infection. To measure C. parvum growth in infected monolayers, the medium in the top compartment was removed, and 50 µl of buffer (5 mM Tris-HCl [pH 8.5]) containing 50 µg/ml proteinase K (Sigma) was added. Cells were scraped into the lysis buffer using a pipette tip, and the lysate was transferred to a PCR tube and incubated at 37°C for 60 min, 56°C for 60 min, and 95°C for 10 min. Two microliters of the lysate was used as a template in the quantitative PCRs (qPCRs) with SYBR green PCR master mix (Applied Biosystems). Reactions were performed on a Stratagene MX3000P quantitative reverse transcription-PCR (qRT-PCR) system with the following amplification conditions: initial denaturation at 95°C for 10 min and 45 cycles of denaturation at 95°C for 5 s, annealing at 55°C for 10 s, and extension at 72°C for 30 s. The sequences of the primers targeting C. parvum GAPDH (glyceraldehyde-3-phosphate dehydrogenase) are as follows: forward primer 5′ AAGGACTGGAGAGCAGGAAG 3′ and reverse primer 5′ AAAGCTGGGATGACCTTACC 3′. A standard curve for C. parvum genomic DNA was generated by lysing a known number of oocysts and creating a dilution series.

### Antigen preparation.

About 48 h postinfection, medium in the top compartment of the infected Transwells was removed. Groups of 10 Transwells were lysed in 100 µl of NP-40 lysis buffer (150 mM sodium chloride, 1% NP-40, 50 mM Tris [pH 8.0]) with protease inhibitor cocktail (Roche Diagnostics). The protein concentration of the lysate was checked by bicinchoninic acid (BCA) assay (Thermo Scientific), adjusted to 1 mg/ml, and stored at −20°C. Antigen was emulsified with TiterMax classic adjuvant (Sigma) in a 1:1 ratio prior to injection into mice.

### Mouse immunization.

Five 8- to 10-week-old female inbred BALB/c mice (Charles River Laboratories, Inc.) were immunized with 6 injections of antigen over 2 weeks. Mice were injected in the same footpad every 3 or 4 days. The first two injections consisted of a 1:1 emulsion of antigen and adjuvant, and the remaining 4 injections consisted of antigen only. Each injection was 20 µl in volume: when using antigen-adjuvant mixture, the mice received 10 µg of antigen, and when using antigen alone, the mice received 20 µg of antigen. Seventeen days after the initial injection, the mice were sacrificed and the popliteal lymph nodes draining the injected footpads were isolated and kept in serum-free medium on ice until fusion.

### Hybridoma fusion and screening.

The popliteal lymph node cells were fused with myeloma cells (P3X63Ag8.653) at a 5:1 ratio with polyethylene glycol 1500 (PEG 1500; Sigma-Aldrich) following standard procedures by the Washington University Hybridoma Center (43). Supernatants were harvested for screening when the cells were 50% confluent (about 2 weeks after fusion). Hybridomas were screened for reactivity against C. parvum grown in Caco-2 cells plated on 96-well plates (Greiner Bio-One). Monolayers cells were permeabilized with 0.05% saponin (Sigma) and blocked with a solution containing 0.05% saponin, 5% normal goat serum, and 5% FBS. Hybridoma supernatants were added to the wells in addition to a solution containing anti-RH antibody (rabbit polyclonal sera raised against Toxoplasma gondii strain RH; Covance WU 1047) at a 1:1,000 dilution and 0.02% saponin. After a 1-h incubation, cells were washed and then stained with secondary antibodies conjugated to Alexa Fluor dyes (Thermo Fisher) diluted in 0.01% saponin solution. Positive hybridomas were expanded in Iscoves’ medium (Sigma) supplemented with 20% FBS, cryopreserved in culture medium supplemented with 10% dimethyl sulfoxide (DMSO), and stored in liquid nitrogen.

### Hybridoma subcloning and expansion.

Positive hybridomas were taken through two rounds of subcloning by single-cell limiting dilution to ensure clonality. At each stage, the hybridoma suicide supernatants (collected when overgrowth of cells results in a decrease in viability to 20%) were screened for reactivity. Hybridomas were weaned in a stepwise manner into 10% FBS before being transferred into a CELLine flask (Wheaton) for concentrated monoclonal antibody production. The isotype identification for the hybridomas was done with the Pierce Rapid isotyping kit (Thermo Fisher). Some of the hybridoma isotyping results were confirmed with the Hybridoma Core at Washington University or the Rapid mouse immunoglobulin isotyping kit (Antagen).

### Immunofluorescence analysis of C. parvum in mIECs.

mIECs grown on Transwells were infected with 1 × 10^6^ oocysts 24 h after plating. After 4 h, monolayers were washed with DPBS and the medium in the top Transwell compartment was replaced. Monolayers were fixed 24 h postinfection, permeabilized, and blocked as described above. Both primary antibodies (hybridoma supernatants listed in Table 1) and secondary antibodies (anti-IgG or anti-IgM) conjugated to Alexa Fluor dyes (Thermo Fisher) were diluted in 0.01% saponin solution for staining. Samples were stained with Hoechst (Thermo Fisher), and the membrane was cut out from the Transwell insert and mounted with Prolong Diamond antifade mountant (Thermo Fisher). Imaging was done on a Zeiss Axioskop Mot Plus fluorescence microscope equipped with a 100×, 1.4 NA Zeiss Plan Apochromat oil objective and an AxioCam MRm monochrome digital camera. Exposure times for each antibody were established based on optimal autoexposure times for the most intensely staining stages and then maintained consistently for all stages for a given antibody. Images were acquired using AxioVision software (Carl Zeiss, Inc.).

### Immunofluorescence analysis of C. parvum in HCT-8 cells.

HCT-8 cells grown on coverslips were infected 24 h postseeding with 1 × 10^6^ oocysts per well and fixed at 24 h postinfection for asexual stages or 48 to 72 h postinfection for sexual stages and then stained for immunofluorescent antibody (IFA) as described above. To identify type II meronts, cells were infected with 1 × 10^6^ excysted oocysts per well and then incubated with 10 µM EdU for 2 h starting 30 h postinfection before fixation and permeabilized as described above. Cells were then treated with the Click-iT Plus EdU Alexa Fluor 488 imaging kit (Thermo Fisher) for 30 min and then stained with mouse monoclonal antibodies and rabbit anti-RH to detect C. parvum, followed by secondary antibodies conjugated to Alexa Fluor dyes (Thermo Fisher). Cells were stained with Hoechst and mounted as described previously. To detect individual microgametocyte nuclei, infected HCT-8 cells were either stained with anti-phosphohistone H3 (Cell Signaling Technology; 1:200) or treated with 10 µM EdU for 2 h starting 46 h postinfection before fixing and staining as described above. To detect host actin, infected HCT-8 cells were fixed and permeabilized as described above and then incubated with Alexa Fluor 488-phalloidin (Thermo Fisher) for 20 min before proceeding with antibody staining, as described above. Epifluorescent images were acquired as described for the mIECs. For confocal images, infected monolayers were prepared as described and then viewed with a Zeiss LSM880 laser scanning confocal microscope (Carl Zeiss, Inc.) equipped with a 63×, 1.4 NA Zeiss Plan Apochromat oil objective and a GaAsP detector. ZEN 2.1 Black Edition software was used to obtain z-stacks through the entire height of the parasites with confocal z-slices of 0.230 µm. Three-dimensional images were generated using the visualization module of Volocity version 6.3 (Improvision).

### Western blot analysis.

Oocysts and sporozoites were lysed in 1% NP-40 lysis buffer containing protease inhibitor cocktail (Roche Diagnostics). Oocysts were bleached prior to lysis. Sporozoites were prepared from excysted oocysts that were filtered through a 1-µm-pore filter (Whatman) to remove unexcysted oocysts and excysted oocyst shells. Samples were lysed on ice for 30 min with occasional vortexing and then centrifuged at 14,000 for 3 min; the supernatant was moved to a new tube and frozen at −20°C. Prior to use, lysates were thawed and reduced using dithiothreitol (DTT) at a final concentration of 100 mM. Samples were resolved on a 10% SDS-PAGE gel and then transferred to a nitrocellulose membrane, blocked with the Odyssey blocking buffer (LI-COR). Anti-RH antibody was used at a concentration of 1:500. A secondary goat anti-mouse IR dye 800CW (LI-COR) was used at a concentration of 1:10,000. Samples were imaged with a LI-COR Odyssey imaging system.

For detection with monoclonal antibodies, sporozoite samples obtained after oocyst excystation were lysed in sample buffer and boiled for 5 min at 95°C. They were then centrifuged at 14,000 × *g* for 1 min, and the supernatant was used for loading. Samples were resolved on a 10% SDS-PAGE gel, transferred to a nitrocellulose membrane, and blocked with 5% BSA. Monoclonal antibodies against C. parvum were used at 1:250, and secondary goat anti-mouse antibody conjugated to horseradish peroxidase (HRP) was used at 1:10,000. Signal detection was done using the Amersham ECL Prime Western kit (GE Healthcare). Among the antibodies highlighted here, we were only able to detect reliable signals in sporozoites for two of them (i.e., 1B5 and 1A5).

### Transmission electron microscopy.

For ultrastructural analyses, infected mIECs on Transwell membranes or trypsinized HCT-8 monolayers were fixed in a freshly prepared mixture of 1% glutaraldehyde and 1% osmium tetroxide (both from Polysciences, Inc.) in 50 mM phosphate buffer at 4°C for 30 min. Samples were then rinsed multiple times in cold distilled water (dH_2_O) prior to en bloc staining with 1% aqueous uranyl acetate (Ted Pella, Inc.) at 4°C for 3 h. Transwell membranes were removed from the insert using a scalpel. Following several rinses in dH_2_O, samples were dehydrated in a graded series of ethanol and embedded in Eponate 12 resin (Ted Pella, Inc.). Sections of 95 nm were cut with a Leica Ultracut UCT ultramicrotome (Leica Microsystems, Inc.), stained with uranyl acetate and lead citrate, and viewed on a JEOL 1200 EX transmission electron microscope (JEOL United States, Inc.) equipped with an AMT 8 megapixel digital camera and AMT Image Capture Engine V602 software (Advanced Microscopy Techniques).
