# Supplementary figures and images for: Monoclonal Antibodies to Intracellular Stages of Cryptosporidium parvum Define Life Cycle Progression In Vitro
Source: mSphere. 2018 May 30;3(3):e00124-18. doi: 10.1128/mSphere.00124-18 (PMC5976880; doi:10.1128/mSphere.00124-18)

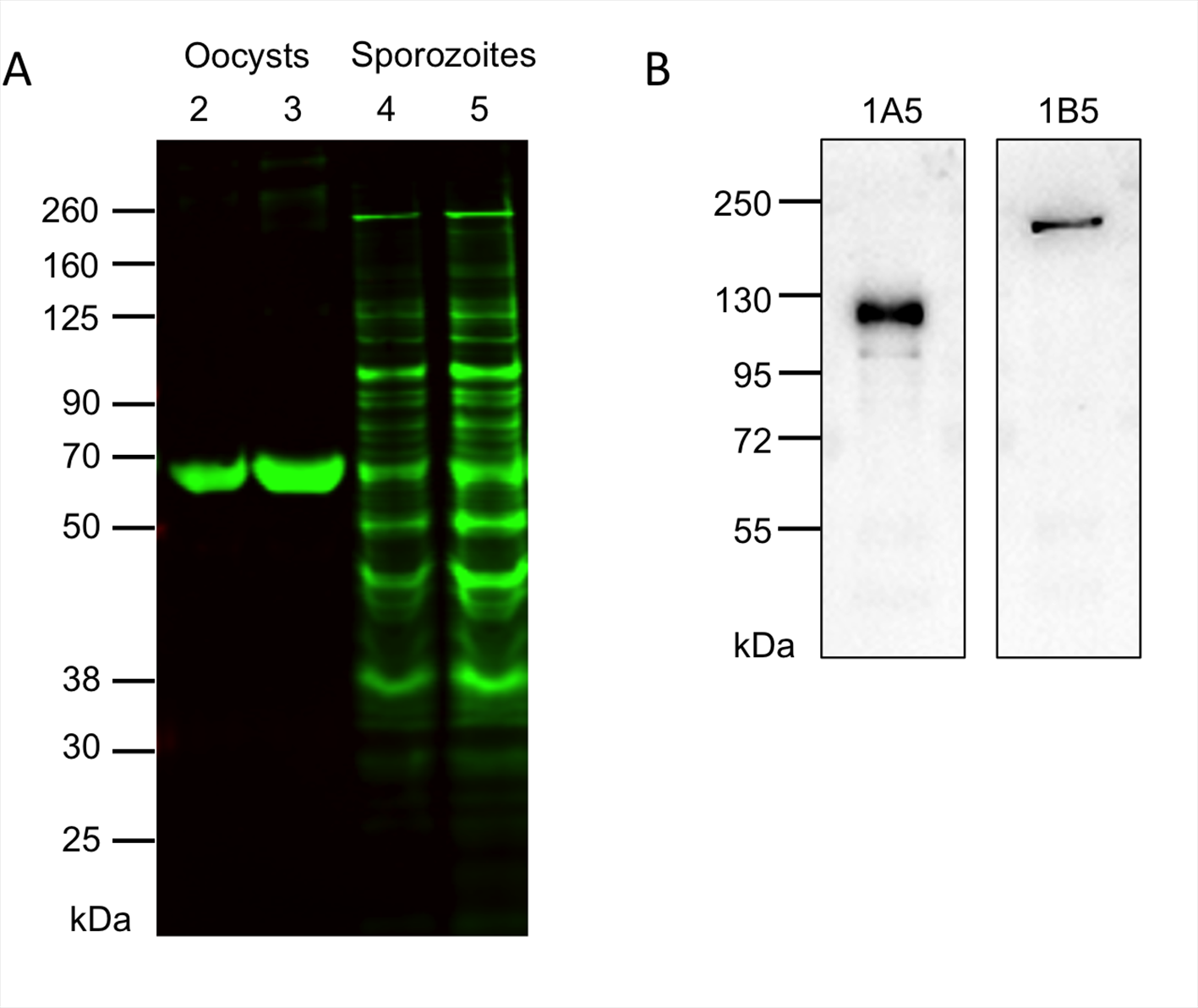

Supplement: FIG S1 [file sph003182555sf1.tif]

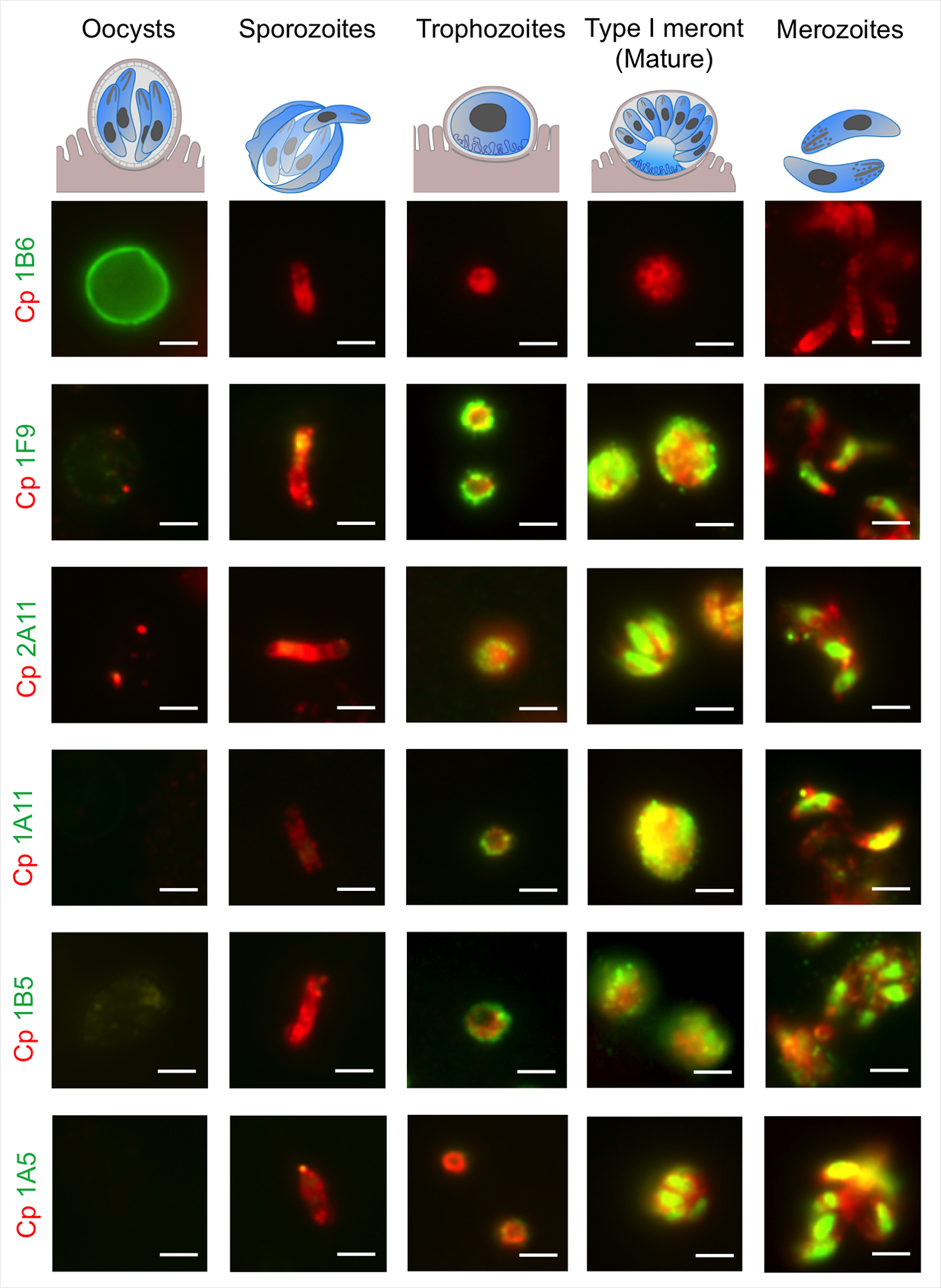

Supplement: FIG S2 [file sph003182555sf2.tif]

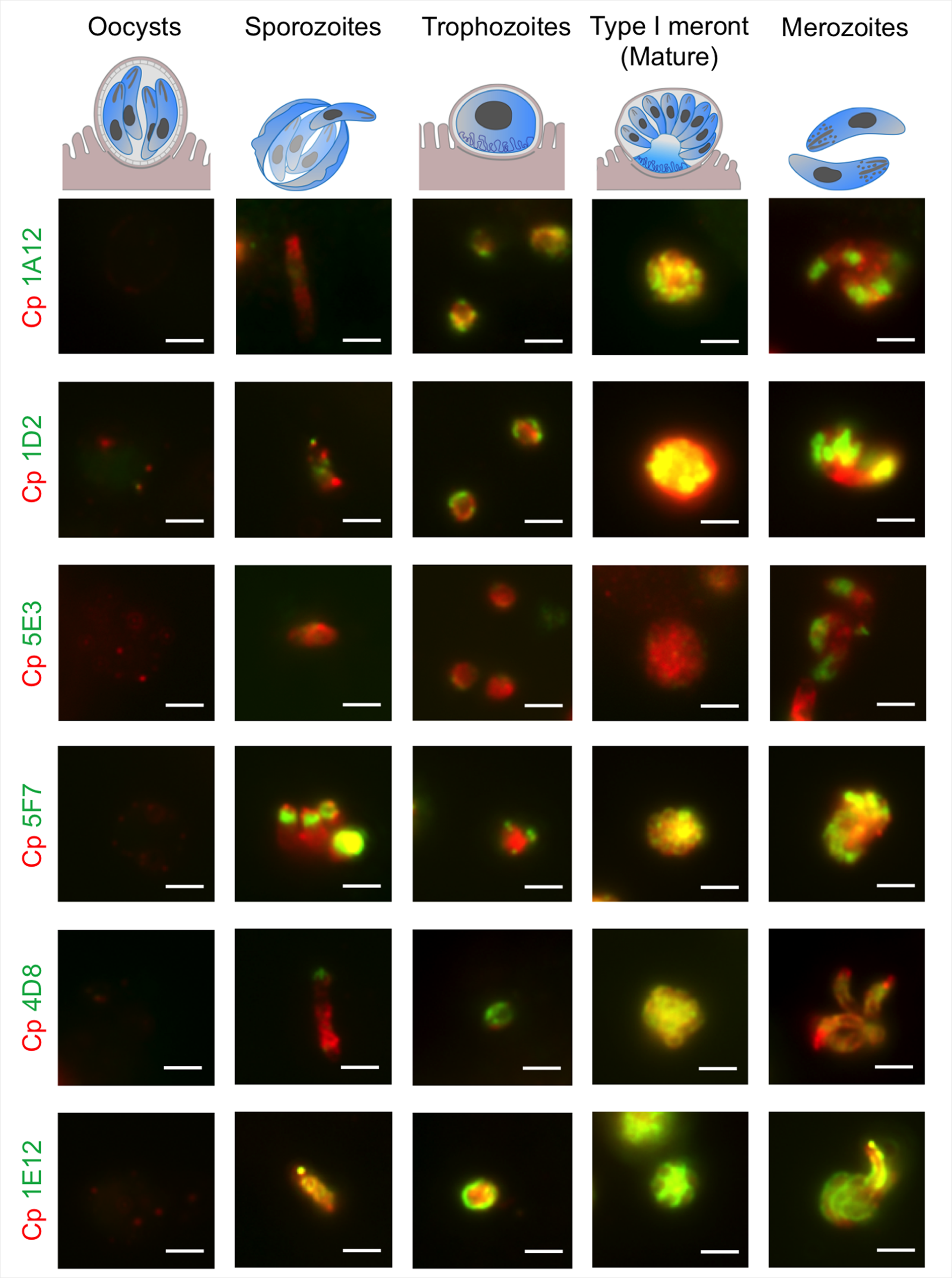

Supplement: FIG S3 [file sph003182555sf3.tif]

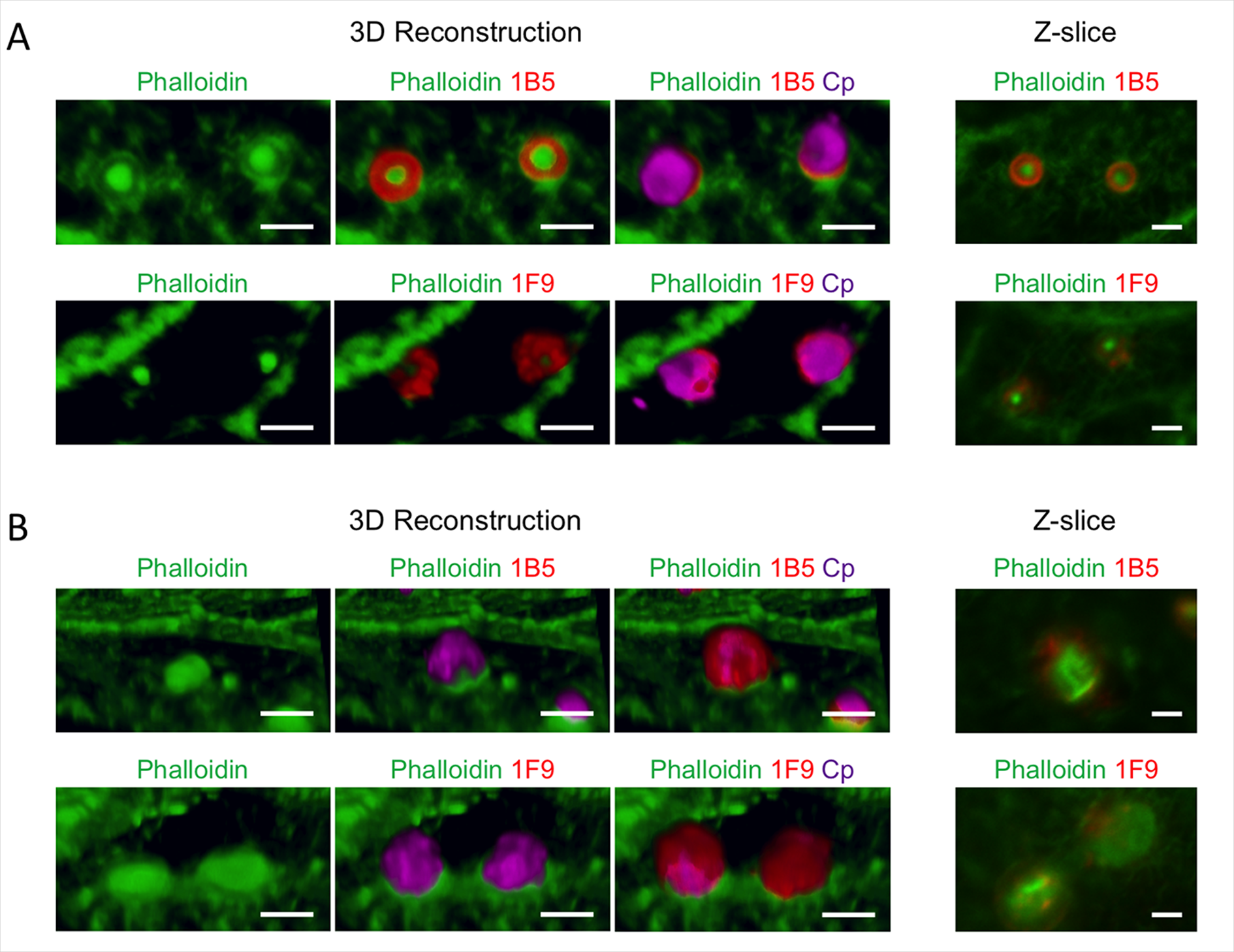

Supplement: FIG S4 [file sph003182555sf4.tif]

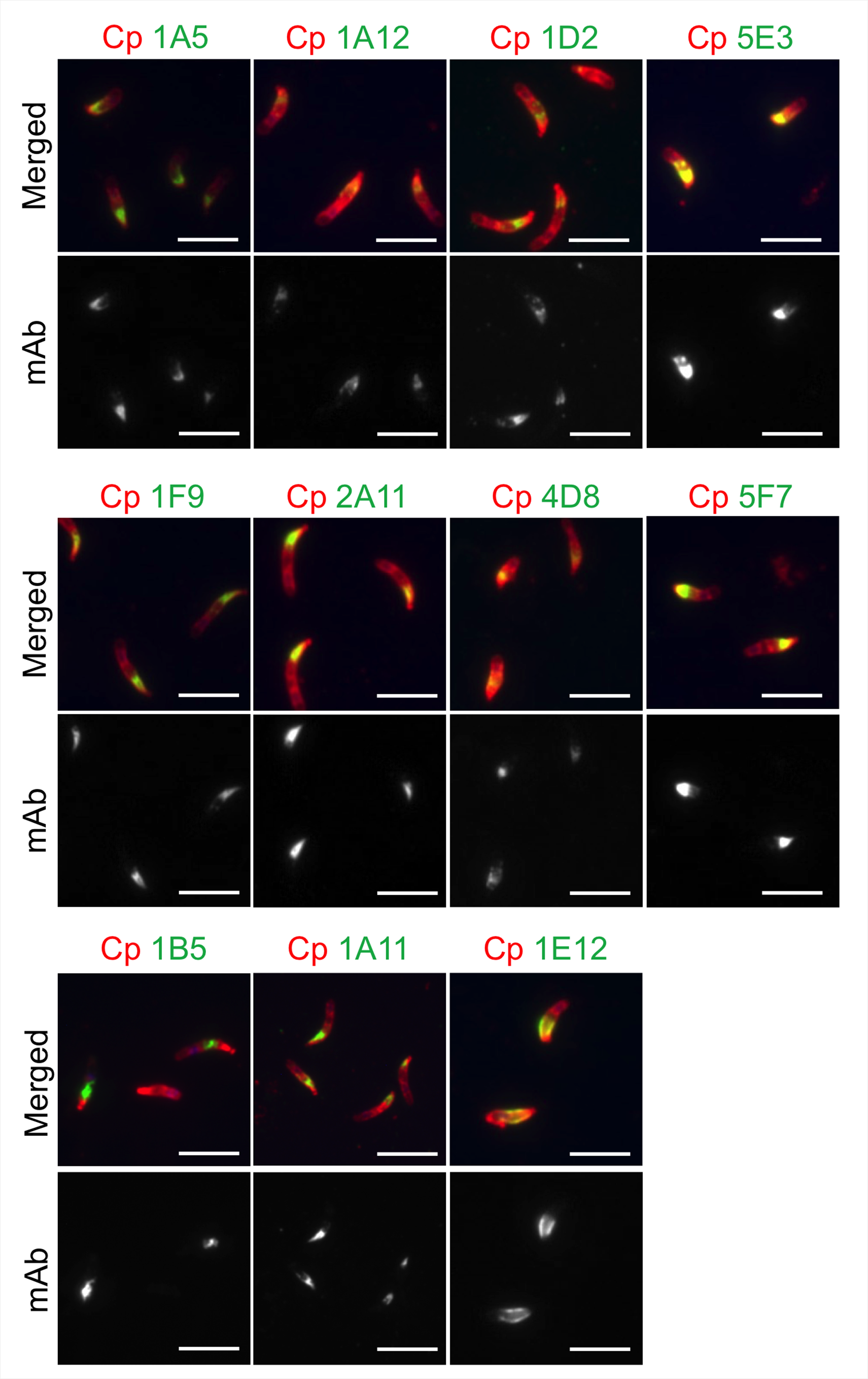

Supplement: FIG S5 [file sph003182555sf5.tif]
